# Selective cycloaddition of ethylene oxide to CO_2_ within the confined space of an amino acid-based metal–organic framework†

**DOI:** 10.1039/d3dt01984e

**Published:** 2023-11-13

**Authors:** Cristina Bilanin, Paula Escamilla, Jesús Ferrando-Soria, Antonio Leyva-Pérez, Donatella Armentano, Emilio Pardo

**Affiliations:** a Instituto de Tecnología Química (UPV–CSIC), Universitat Politècnica de València–Consejo Superior de Investigaciones Científicas Avda. de los Naranjos s/n 46022 Valencia Spain; b Departament de Química Inorgànica, Instituto de Ciencia Molecular (ICMOL), Universitat de València 46980 Paterna València Spain; c Dipartimento di Chimica e Tecnologie Chimiche, Università della Calabria 87036 Cosenza Italy

## Abstract

Host–guest chemistry within the confined space of metal–organic frameworks (MOFs) offers an almost unlimited myriad of possibilities, hardly accessible with other materials. Here we report the synthesis and physical characterization, with atomic resolution by single-crystal X-ray diffraction, of a novel water-stable tridimensional MOF, derived from the amino acid *S*-methyl-l-cysteine, {SrZn_6_[(*S*,*S*)-Mecysmox]_3_(OH)_2_(H_2_O)}·9H_2_O (1), and its application as a robust and efficient solid catalyst for the cycloaddition reaction of ethylene/propylene oxide with CO_2_ to afford ethylene/propylene carbonate with yields of up to 95% and selectivity of up to 100%. These results nicely illustrate the great potential of MOFs to be game changers for the selective synthesis of industrially relevant products, representing a powerful alternative to the current heterogeneous catalysts.

## Abstract 2

We report a novel bio-friendly water-stable tridimensional MOF with application as a solid catalyst for the cycloaddition reaction of ethylene oxide.

## Graphical abstract

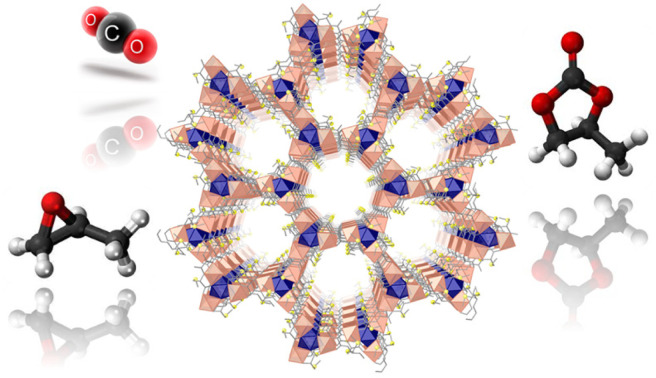

## Introduction

Metal–organic frameworks (MOFs)^1,2^ have been featured among porous materials owing to their particular and tailorable host–guest chemistry.^3,4^ This, for example, has enabled them to outperform state-of-the-art materials in different applications, such as heterogeneous catalysis, where molecular recognition interactions of guests are of paramount importance to achieve high conversion and selectivity.^5,6^ This success is, in part, a direct consequence of the possibility in MOFs to precisely tune the size, shape and functionality of the MOF pores,^7,8^ which offers the opportunity to have the appropriate orientation and proximity of reactants to lead to non-default products, as well as stabilize reaction intermediates, which otherwise could not be achieved.^9,10^

The cycloaddition of ethylene oxide (EO) to CO_2_ to give ethylene carbonate (EC) is a fundamental industrial reaction, since EC finds application as a general solvent in fine chemistry and as an electrolyte in lithium-based batteries, and is a key intermediate for the synthesis of other industrial chemicals, *i.e.* ethylene glycol (Shell Omega process).^11^ Industrial synthesis employs homogeneous catalysts, although it is recognized that solid catalysts will enable the implementation of a more practical and environmentally benign process.^12^ This is particularly convenient when managing an extremely hazardous reactant such as EO.^13^ However, the number of studies in the open literature dealing with solid-catalyzed cycloadditions of EO to CO_2_ is scarce; there are <30 scientific open publications according to a database searching for this specific reaction, and most of them have been published in the last 10 years (see Fig. S1 in the ESI†).^14–17^ This number gets more surprising when compared to the number of studies devoted to the synthesis of propylene carbonate (PC), which, despite being relatively less used in industry than EC, is reported in >250 publications (one order of magnitude higher with the same database searching).^18–21^ Thus, more studies on the synthesis of EC with solid catalysts seem timely and necessary.

Here, we report a novel bio-friendly water-stable tridimensional (3D) MOF, derived from the amino acid *S*-methyl-l-cysteine, with the formula {SrZn_6_[(*S*,*S*)-Mecysmox]_3_(OH)_2_(H_2_O)}·9H_2_O (1) (Mecysmox = bis[*S*-methylcysteine]oxalyl diamide), and its application as a solid catalyst for the cycloaddition reaction of EO to CO_2_. The very small (0.6 nm) diameter size and unique environment of the MOF pores enable the selective synthesis of EC without competitive hydrolysis^12^ and polymerization,^22^ even using water as a solvent. Besides, the constrained catalytic site of MOF 1 makes propylene oxide (PO) react like EO, although the former is usually ten times less reactive than the latter (see ahead for discussion). This amino acid-based solid provides an alternative solution to some of the scientific problems in this field since the new catalyst is cheap and easy to prepare and efficiently catalyzes the reaction not only of EO but also of the industrially-relevant PO substrate.

## Results and discussion

### Synthesis and characterization

**Fig. 1:**
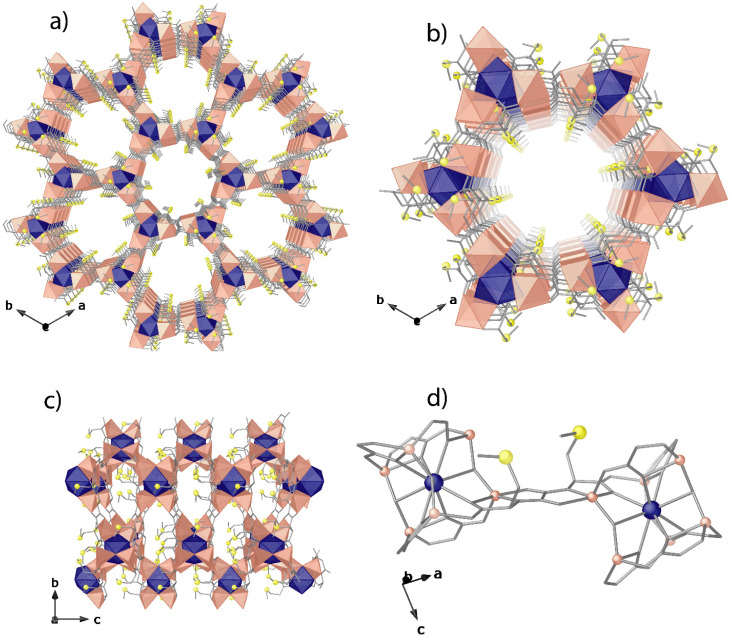
Perspective views of the porous networks of 1 (a) and a single channel along the *c* (a and b) and *a* (c) axes. Perspective view of the {Zn^II^_2_[(*S*,*S*)-Mecysmox]} dimer connecting Sr^II^ cations (d). Colour code: Zn, Sr and S atoms are represented by salmon polyhedra, blue polyhedra, and yellow spheres, respectively, whereas the ligands (except sulphur) are depicted as gray sticks.

**Fig. 2:**
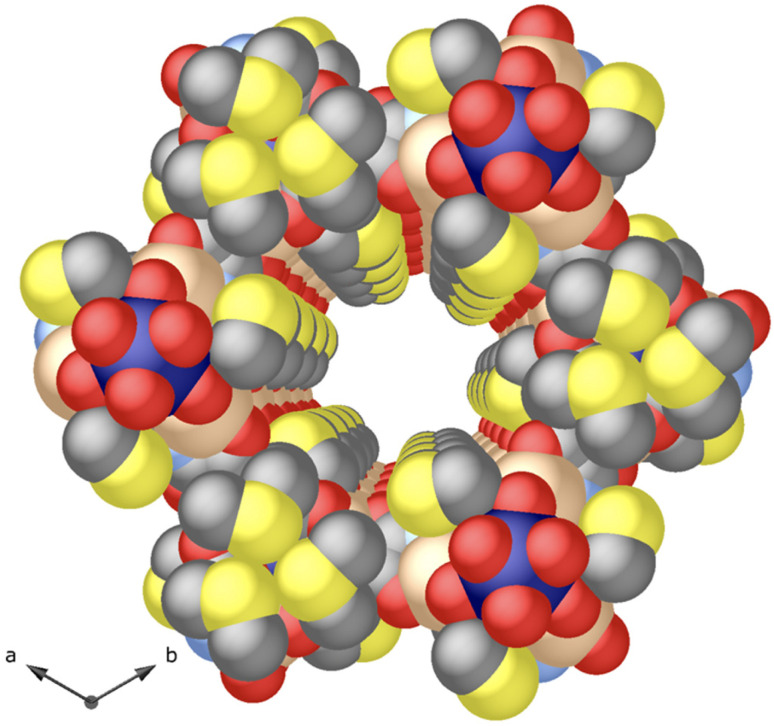
View of one single pore of 1 along the *c* crystallographic axis showing the porous structure in the space filling model (van der Waals radii). Zn(ii) and Sr(ii) metal ions are represented by salmon and blue spheres, respectively, whereas the oxygen, carbon, nitrogen and sulphur atoms from the ligands are depicted as red, grey, light blue and yellow spheres.

Compound 1 was synthesised as colourless hexagonal prisms with a slow diffusion technique (see the Experimental section, ESI†). The crystal structure of 1 could be determined by single-crystal X-ray diffraction (SC-XRD) (see the ESI† for structural details and refinement). 1 crystallizes in the chiral *P*6_3_ space group (Table S1, ESI†), presenting a honey-comb chiral 3D strontium(ii)–zinc(ii) network (Fig. 1). 1 exhibits a uninodal acs chiral net built by strontium(ii) vertexes and *trans*-oxamidato-bridged zinc(ii) units, {Zn^II^_2_[(*S*,*S*)-Mecysmox]} (Fig. 1d and S2, ESI†), which act as linkers between the Sr^II^ ions through the carboxylate groups. The hexagonal channels of *ca*. 0.6 nm are decorated with the functional flexible dimethyl thioether residues of the methylcysteine amino acid (Fig. 1b, 2 and S3†). In the 3D porous structure of 1, the methylcysteine chains show, as detected, one of the two crystallographically distinct moieties in a distended conformation towards the pores and the other one pretty bent, with the terminal methyl groups pointing in smaller interstitial voids developing along the *a* axis (Fig. 1c and S4†). The confined space allows two possible conformations for the distended dimethyl thioether residues of the methylcysteine amino acid, which show statistical disorder and thus have been refined in two different positions/orientations. This is perfectly in line with the outstanding and intrinsic flexibility of the amino acid moieties entirely confined in pores. The network connection involving the *trans*-oxamidato-bridged dizinc(ii) units, {Zn^II^_2_[(*S*,*S*)-Mecysmox]}, and Sr^II^ cations through the carboxylate groups is further assisted by water/hydroxide groups (Fig. 1d).

In 1, the Zn(ii) coordination environment is reminiscent of catalytically active Zn-based compounds. The two crystallographically distinct Zn(ii) metal ions share a bridging water/hydroxide group and reside in an environment describing a trigonal bipyramidal geometry being coordinated by a nitrogen and three oxygen atoms belonging to the Mecysmox ligand and a water/hydroxide bridging group (in a 1 : 2 statistical distribution). The Zn–OH bond length is 1.96(2) and 1.97(2) Å for Zn1 and Zn2, respectively, and represents the shortest distance in the environment. The Zn–N_oxamate_ [1.95(2) and 2.03(2) Å for Zn1 and Zn2, respectively] and the Zn–O_oxamate_ bond lengths [range 1.99(2)–2.22(2) and 2.04(2)–2.24(2) Å for Zn1 and Zn2, respectively] are in agreement with those found in the literature.^23^ The Zn–Zn distance across the hydroxide bridge is 3.71(1) Å.

**Fig. 3:**
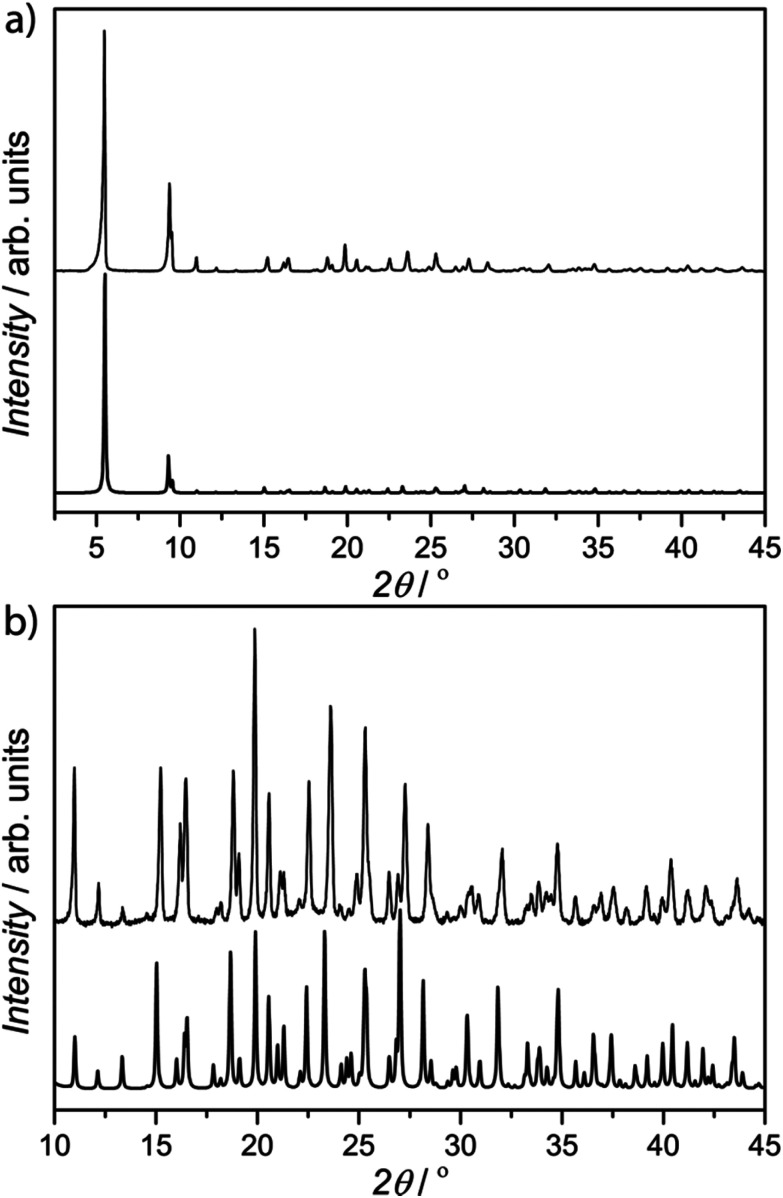
(a) Calculated (bold lines) and experimental (solid lines) PXRD pattern profiles of 1 in the 2*θ* range of 2.0–45.0° at room temperature, and an enlarged image in the range of 10.0–45.0° (b).

In addition, the chemical nature of 1 was also determined using different characterization techniques. In this respect, the atom composition was further established by elemental (C, H, S, and N) analyses (ESI†). Meanwhile, powder X-ray diffraction (PXRD) allowed us to confirm the homogeneity and purity of the bulk sample, by comparison with the theoretical pattern diffraction extracted from the solved crystal structure (Fig. 3 and S5†). The solvent content was validated by thermogravimetric analysis (TGA, Fig. S6†), where it was observed that there was a mass loss of *ca*. 10% that corresponds to 9H_2_O molecules. N_2_ adsorption isotherms at 77 K revealed a permanent microporosity in 1 (Fig. S7†).

### Catalytic studies

The novel solid material 1 was tested as a catalyst (6 mol%) for the cycloaddition reaction of ethylene oxide (EO) (2) with CO_2_ at 120 °C for 4 h, using tetraoctylammonium bromide (TOAB, 2 mol%) as a catalytic source of bromide anions under a CO_2_ pressure of 25 bar. These reaction conditions are typical for ZnBr_2_ and ionic liquids.^24,25^ However, we introduce here the use of less expensive and more soluble TOAB. The reaction was analysed by gas chromatography (GC), after cooling and venting out the reactor and diluting with THF containing *n*-decane as an external standard. As it can be seen in Table 1, the desired product ethylene carbonate (EC) (3) was obtained in 95% yield with complete selectivity under the indicated catalytic reaction conditions (entry 1). The amount of TOAB could be reduced to half, but the yield of 3 significantly decreased (68%), and a much lower yield was obtained with a typical ionic liquid as a bromide source (*i.e. N*,*N*′-*n*-butylmethylimidazolium bromide, bmimBr, 47% with 4 mol%), which showcases the use of TOAB as a new source of anion in this reaction. Some control experiments were performed and the results were as follows: (1) reaction with only ZnBr_2_ (neither MOF nor TOAB): 3.5%; (2) reaction with only TOAB (neither MOF nor ZnBr_2_): 12%; (3) reaction with an MOF ligand (neither MOF nor ZnBr_2_): 25.3%; and (4) reaction with an MOF ligand + ZnBr_2_ (no MOF): 77.5%. These results demonstrate the need for supporting Zn in the MOF to efficiently catalyse the reaction.

**Table 1:** Catalytic results for the cycloaddition reaction of ethylene oxide (EO 2) with CO_2_ within MOF 1. GC results, products are identified by GC-MS and NMR. TOAB: tetraoctylammonium bromide (the reaction does not proceed without TOAB). Selectivity is 100% for the indicated products; other by-products were not detected

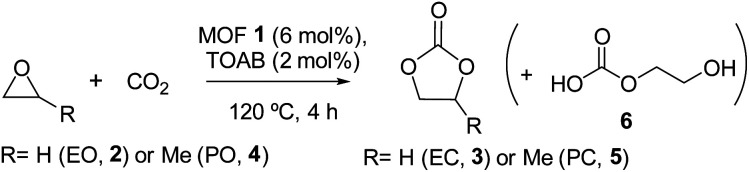				
Entry	Reactant^a^	Solvent (1.6 M)	CO_2_ (bar)	Product (yield, %)
1	2	THF	25	3 (95) [68]^b^
2	4			5 (94)
3	2 + 4			3 (50), 5 (45)^c^
4	2		30	3 (94), 6 (6)
5			5	3 (83)^d^
6	2/H_2_O	THF-*d*^4^	25	3 (66), 6 (34)
				3 [56], 6 [44]^e^
7	2/MeOH	THF		3 (91), 6 (5)
8	2/NaOH			3 (68)
9	2/SMe_2_			3 (42)
10	2/HSEt			3 (18), 7 (46)^f^
11	3			6 (20)^g^

a 3 equivalents of co-reactant to EO 2.

b Between brackets is the result for 1 mol% of TOAB; a similar result was obtained with *N*,*N*′-butylmethylimidazolium bromide (bmimBr).

c 50 mol% of each reagent.

d 25 bar of N_2_.

e Between brackets is the result with 30 equivalents of water.

f 7 is the corresponding thioketal (see Fig. S11†).

g 1 M solution, product 6 was not detected without CO_2_ pressure.

The kinetic radii of EO 2 and EC 3 are 0.21 and 0.24 nm, respectively, calculated by molecular mechanics (MM2) after energy minimization in the vacuum. Thus, both molecules can diffuse across MOF 1's pores to reach the catalytic Zn(ii) sites, together with CO_2_ and Br^−^ (this MOF family accepts anions within the pores).^26,27^ Following this rationale, the kinetic radii of propylene oxide (PO) (4) and propylene carbonate (PC) (5) were also calculated and found to be 0.32 and 0.38 nm respectively (MM2 calculations). Thus, there is the possibility of diffusion across the MOF channels. Indeed, the reaction of 4 to 5 proceeds under the same reaction conditions with a similar high yield (94%, entry 2). When both reactants 2 and 4 were co-fed in the reaction (50 mol% mixture), both products 3 and 5 were nearly quantitatively formed in equimolecular amounts (entry 3). Taking into account that PO 4 is usually less reactive than EO 2 due to steric reasons,^12^ the results here suggest that the constrained environment of the catalytic MOF site enables the good activation of 4 towards CO_2_ cycloaddition.

In order to confirm the substrate/product selectivity exerted by the MOF, we also tested three larger epoxides as reactants for the cycloaddition reaction. The alkenes were α-pinene, (+)limonene and cyclohexene. Any conversion was only found for the latter, with 41.7% of the cyclohexene oxide product (complete selectivity). These results nicely confirm the size discrimination exerted by the MOF.

**Fig. 4:**
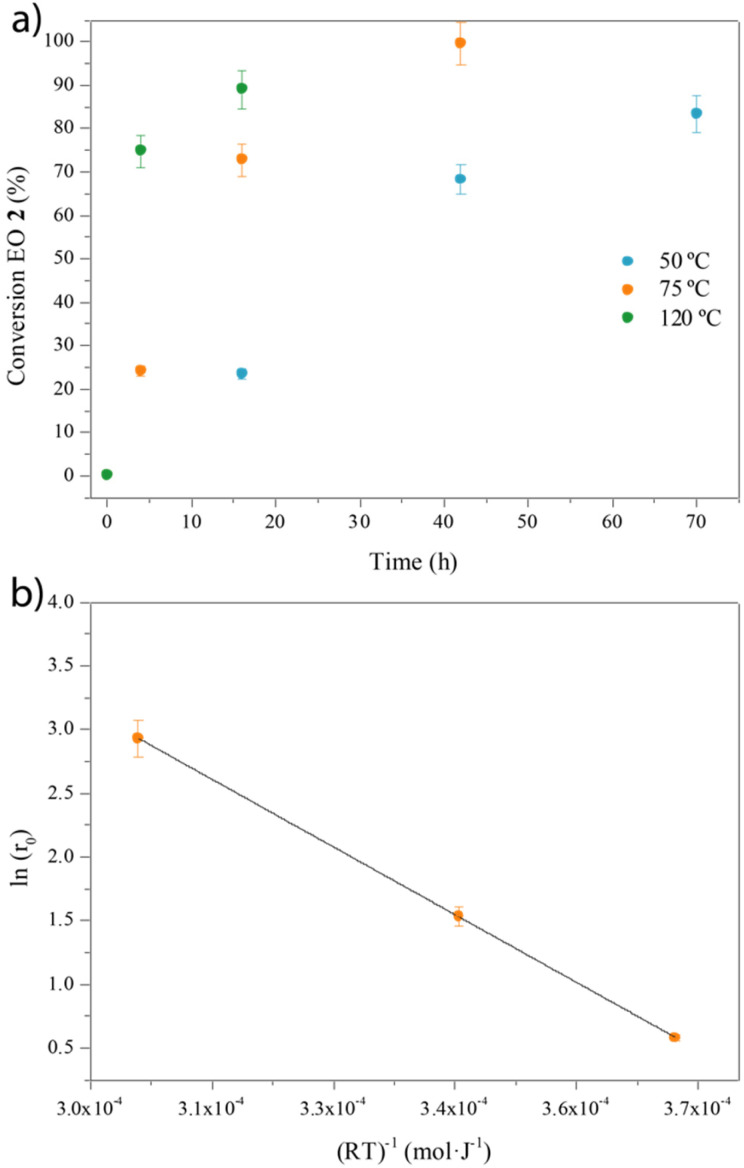
(a) Kinetic plots for the cycloaddition reaction of ethylene oxide (EO 2) dissolved in THF (3.3 M) to give ethylene carbonate (EC 3) with catalytic amounts of MOF 1 (6 mol%) and TOAB (2 mol%) under 25 bar of CO_2_ and at different reaction temperatures. Selectivity is 100%. (b) Arrhenius plot to calculate the activation energy of the reaction: 
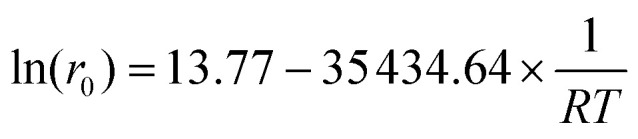
; *E*_a_ = 8.47 kcal mol^−1^; *R*^2^ = 0.99992. Error bars account for 5% uncertainty.

The CO_2_ pressure finds a plateau at *ca*. 30 bar (94%, entry 4; see also Fig. S8†); however, the use of just 5 bar of CO_2_ together with 25 bar of N_2_ yields a reasonable 83% of EC 3 (entry 5). It should be noted here that under this pressure, the reaction without MOF 1 proceeds with a yield of just 32%. Kinetic studies show that high yields of EC 3 are still obtained after just 4 h of reaction time at 120 °C (Fig. S9†) and that the reaction temperature can be decreased down to 50 °C with >80% yields of 3 at prolonged reaction times (Fig. 4a). Overall, these results show that the cycloaddition reaction of EO 2 with CO_2_ catalyzed by the Zn(ii) MOF 1 can be performed with high yields and complete selectivity to EC 3 within a wide range of conditions, including with and without solvent, CO_2_ pressures between 5 and 30 bar and reaction temperatures between 50 and 120 °C. These results should also be applicable to PO 4 and PC 5. Remarkably, the activation energy of the MOF 1-catalyzed reaction, calculated from the corresponding Arrhenius plot, is 8.4(7) kcal mol^−1^ (Fig. 4b) within the range of many homogeneous catalysts^28,29^ and much lower than the best solid catalysts reported,^30^ as far as we know.

The desired product EC 3 is very stable under any reaction conditions employed and only partially evolves to a secondary product (entry 6), which is characterized by GC coupled to mass spectrometry (GC-MS) and ^1^H, ^13^C and distortionless enhancement by polarization transfer (DEPT) NMR, employing THF-*d*^4^ as the reaction solvent without isolation, suggesting that it could be the rarely detected compound 6, the postulated intermediate in the hydrolysis of EC 3 to ethylene glycol. It can be seen that up to a 44% yield of 6 can be obtained when using up to 30 equivalents of H_2_O with respect to EO 2, without any trace of ethylene glycol or polymerized products in the reaction medium. The amount of intermediate 6 increases with the amount of water present in the reaction, from the residual water content in the MOF (see the MOF's formula above and entries 3 and 4 in Table 1) to 30 equivalents (entry 6). Conversely, different competing nucleophiles such as MeOH (entry 7), NaOH (entry 8) and MeSMe (entry 9) inhibit its formation. The use of EtSH as a nucleophile leads to the formation of the corresponding thioketal 7 (entry 10), also characterized by GC-MS and NMR, which is formed through a similar reaction pathway to 6 (Fig. S10†). EC 3 could also be used as a reactant to give 20% of 6, with MOF 1 as the catalyst under 25 bar of CO_2_ (entry 11); otherwise 6 is not formed. These results, together, strongly suggest that the particular reactive environment provided by the water-tolerant Zn(ii) MOF 1^31^ not only stabilizes product EC 3 but also activates water molecules,^32,33^ and under a pressurized CO_2_ atmosphere, it enables the formation and detection of product 6 and the complete inhibition of ethylene glycol formation.

**Fig. 5:**
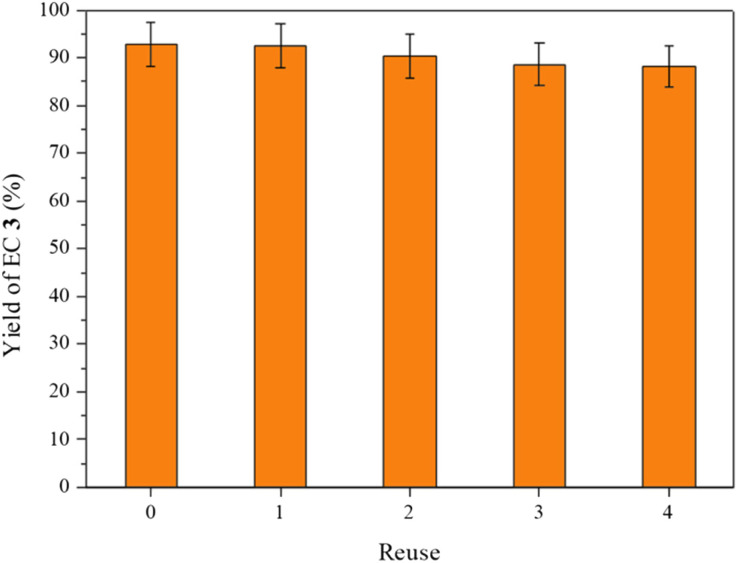
Reuses of MOF 1 (6 mol%) as a catalyst for the cycloaddition reaction of ethylene oxide (EO, 2) dissolved in THF (1.6 M) to give ethylene carbonate (EC, 3) with TOAB (2 mol%) under 25 bar of CO_2_ at 120 °C for 4 h. Error bars account for 5% uncertainty.

A hot filtration test for MOF 1 shows that the catalytically active species are not leached out into the solution under the reaction conditions employed (Fig. S11†). Indeed, the solid catalyst could be reused up to five times to give a similar high yield of EC 3 (*ca*. 90%, Fig. 5). Analysis of the reused MOF 1 by PXRD does not show any erosion of the MOF structure, thus the Zn(ii) sites must still be active (Fig. S5c†). However, a N_2_ adsorption isotherm of the reused solid shows a sizeable decrease in porosity, probably due to a strong adsorption of products (Fig. S7†). Fourier-transform infrared (FT-IR) spectroscopy measurements of the used solid catalyst confirm the stability of the solid and the presence of some adsorbed organic products in the MOF (Fig. S12†). The adsorption capacity of a microporous material for a relatively complex organic compound like product 3 (*i.e. ca*. 100 Da) can be ∼5 wt%; this value is very typical in MOFs and zeolites, which will explain the 95% yield found in solution. Such a value of the adsorbed material (∼5 wt%) is also enough to produce a 10–15% decrease in the nitrogen adsorption value of the used MOF. The water stability of 1 was confirmed by PXRD studies after 24 hours of immersion in normal water and water at pH = 5 and 11 (Fig. S13†). These results, together, indicate that the MOF catalyst is recoverable and reusable despite some organic products remaining in the solid, probably due to a hindered diffusion through the pores, which was reasonably expected for a microporous solid with such a very small pore.

## Experimental

### Materials and methods

Reagents were obtained from commercial sources and used without further purification unless otherwise indicated. Anhydrous solvents were obtained from resin-exchanger apparatus. Reactions were performed in conventional round-bottomed flasks or sealed vials equipped with a magnetic stirrer. All the products were characterized by gas chromatography-mass spectrometry (GC-MS), proton (^1^H), carbon (^13^C) and distortionless enhancement by polarization transfer (DEPT) nuclear magnetic resonance (NMR) spectroscopy. Gas chromatographic analyses were performed in an instrument equipped with a 25 m capillary column of 1% phenylmethylsilicone using *n*-dodecane as an external standard. GC/MS analyses were performed on a spectrometer equipped with the same column as the GC and operated under the same conditions. ^1^H-, ^13^C and DEPT were recorded in a 300 MHz (or 400 MHz when available) instrument using CD_3_CN as the solvent unless otherwise indicated, containing TMS as an internal standard. Infrared (IR) spectra of the compounds were recorded with a spectrophotometer by impregnating the windows with a dichloromethane solution of the compound and leaving them to evaporate before analysis. H_2_Me_2_-(*S*,*S*)-Mecysmox was prepared following a reported procedure.^34,35^

Elemental (C, H, S, and N) analysis was performed at the Microanalytical Service of the Universitat de València. FT-IR spectra were recorded on a PerkinElmer 882 spectrophotometer as KBr pellets. Thermogravimetric analysis was performed on crystalline samples under a dry N_2_ atmosphere with a Mettler Toledo TGA/STDA 851^e^ thermobalance operating at a heating rate of 10 °C min^−1^.

### Synthetic procedures

#### Synthesis of compound {Sr^II^Zn^II^_6_[(*S*,*S*)-Mecysmox]_3_(OH)_2_(H_2_O)}·9H_2_O (1)

H_2_Me_2_-(*S*,*S*)-Mecysmox^34^ (4.22 g, 12.0 mmol) was suspended in 80 mL of water and treated with a 25% methanolic solution of Me_4_NOH (14.5 mL, 50.0 mmol) until complete dissolution. Then, another aqueous solution (40 mL) containing Sr(NO_3_)_2_ (0.85 g, 4.0 mmol) and Zn(NO_3_)·6H_2_O (6.98 g, 24.0 mmol) was added dropwise under stirring. After further stirring for 10 h, at room temperature, a white polycrystalline powder was obtained and collected *via* filtration and dried with methanol. Yield: 4.83 g, 73%; Anal.: calcd for C_30_H_58_N_6_O_30_S_6_SrZn_6_ (1655.1): C, 21.77; H, 3.53; S, 11.62; N, 5.08%. Found: C, 21.71; H, 3.50; S, 11.65; N, 5.07%. IR (KBr): *ν* = 1602 cm^−1^ (C=O). Well-shaped hexagonal prisms of 1 suitable for X-ray structural analysis could be obtained by slow diffusion, in an H-shaped tube, of H_2_O/DMF (1 : 1) solutions containing stoichiometric amounts of H_2_Me_2_-(*S*,*S*)-Mecysmox (0.021 g, 0.06 mmol) and Me_4_NOH (0.72 mL, 2.5 mmol) in one arm and Sr(NO_3_)_2_ (0.042 g, 0.2 mmol) and Zn(NO_3_)·6H_2_O (0.035 g, 0.12 mmol) in the other. They were isolated by filtration on paper and air-dried. Anal.: calcd for C_30_H_58_N_6_O_30_S_6_SrZn_6_ (1655.1): C, 21.77; H, 3.53; S, 11.62; N, 5.08%. Found: C, 21.73; H, 3.49; S, 11.58; N, 5.06%.

### Single crystal X-ray diffraction

Crystals of 1 with 0.18 × 0.18 × 0.14 mm dimensions were selected and mounted on a MiTeGen MicroMount in Paratone oil and very quickly placed on a liquid nitrogen stream cooled at 100 K to avoid the possible degradation upon dehydration or exposure to air. Diffraction data were collected on a Bruker-Nonius X8APEXII CCD area detector diffractometer using graphite-monochromated Mo-K_α_ radiation (*λ* = 0.71073 Å). The data were processed using SAINT^36^ reduction and SADABS^37^ multi-scan absorption software. The structure was solved with the SHELXS structure solution program using the Patterson method. The model was refined with version 2018/3 of SHELXL against *F*^2^ on all data by full-matrix least squares.^38–40^

In the refinement, all non-hydrogen atoms of the MOF net, except for the highly thermally disordered terminal methyl of the methylcysteine thioether chains from the Mecysmox ligand, were refined anisotropically. The use of some C–C and C–S bond length restraints during the refinement has been reasonably imposed and related to the extraordinary flexibility of methyl thioether chains from the methionine residues, which are dynamic components of the frameworks. Disordered sites for atoms C4S and C4S′ in refinement, belonging to the methylcysteine ligand, result in disorder and have been refined at two different sites. All the hydrogen atoms of the net were set in calculated positions and refined isotropically using the riding model.

The thermal disorder of fragments pointing towards the pores is likely related to the porosity of the network.

The solvent molecules, as normally observed for such porous crystals, were highly disordered and only a few of them were found on the Δ*F* map. The quite large channels featured in this series of MOFs likely account for that.

A summary of the crystallographic data and structure refinement for 1 crystal structure is given in Table S1.† The comments for alerts A and B are described in the CIF using the validation reply form (vrf). The CCDC reference number is 2265848.†

The final geometrical calculations on free voids and the graphical manipulations were carried out with PLATON^41,42^ implemented in WinGX,^43^ and CRYSTAL MAKER^44^ programs, respectively. A cut-off with a 2theta of 56° was applied to the experimental data set.

### Gas adsorption

The N_2_ adsorption–desorption isotherms were carried out at 77 K on polycrystalline samples of 1 before and after catalysis with a BELSORP-mini-X instrument. Samples were first activated with methanol and then evacuated at 348 K for 19 hours under 10^−6^ Torr prior to the analysis.

### X-ray powder diffraction measurements

Polycrystalline samples of 1 before and after catalysis were introduced into 0.5 mm borosilicate capillaries prior to being mounted and aligned on an Empyrean PANalytical powder diffractometer using Cu Kα radiation (*λ* = 1.54056 Å). For each sample, five repeated measurements were collected at room temperature (2*θ* = 2–45°) and merged in a single diffractogram.

### General catalytic procedure

Products 3, 5 and 6 were prepared following the reaction scheme. Catalyst 1 (0.06 eq., 0.16 mmol), reactant 2 or 4 (1 eq., 2.72 mmol) and TOAB (0.02 eq., 0.058 mmol) in 1.7 mL of dry THF were introduced in a reactor equipped with a magnetic stirrer. Then, the reactor was closed, purged with N_2_ two times and carbon dioxide (25 bar) was introduced. The reaction mixture was magnetically stirred in a pre-heated oil bath at 120 °C for typically 4 h. After that time, the resulting mixture was filtered and analyzed by GC, GC-MS and NMR.

### Hot filtration test

Following the general reaction procedure above, the hot reaction mixture was filtered after 30 minutes, when the reaction conversion was approx. 20%. Filtrates were placed into a reactor, which was closed and purged with N_2_ twice, and carbon dioxide (25 bar) was introduced. Finally, the reaction was performed at a temperature of 120 °C. The filtrates were periodically analysed by GC, comparing the results obtained with the solid catalyst still in.

### Reuses

When the reaction was complete, the solid was separated by centrifugation and washed with THF (three times) to remove the ammonium salt and any soluble products. Then, the solid catalyst was dried under vacuum, weighed, and directly used in the next reaction.

## Conclusions

Overall, we have shown that the well-defined MOF 1, characterized with atomic precision by SC-XRD and possessing a very small pore size of *ca*. 0.6 nm, catalyses the cycloaddition reaction of ethylene oxide (EO 2) or propylene oxide (PO 4) with CO_2_ to give EC 3 and PC 5, respectively, with high yields (up to 95%) and selectivity (up to 100%). If water is added to the reaction, even in high excess (30 equivalents), ethylene glycol is not detected but only, tentatively, the elusive hydrolysis intermediate 6 is obtained. These results reveal Zn(ii)-based MOF 1 as a new solid catalyst for the selective synthesis of the industrially relevant products EC 3 and PC 5,^45^ providing an appealing alternative to the current heterogeneous catalysts based on metal-free solids,^16,17,46–48^ ionic liquid-microporous solid composites,^49–52^ and other MOFs,^18,31,45,53–55^ among other catalysts.^56,57^

## Author contributions

C. B. performed and analyzed the catalytic experiments and product characterization. P. E. synthesised 1 and performed the physical characterization. A.L.-P. designed, supervised and wrote the catalytic part. D. A. performed and wrote the crystallographic part. J. F.-S. and E. P. designed, supervised and wrote the manuscript with input from other authors in this work.

## Conflicts of interest

There are no conflicts to declare.

## Supplementary Material

DT-052-D3DT01984E-s001

DT-052-D3DT01984E-s002
