# Supplementary material for: Selective cycloaddition of ethylene oxide to CO2 within the confined space of an amino acid-based metal–organic framework
Source: Dalton Trans. 2023 Nov 13;52(47):18018–26. doi: 10.1039/d3dt01984e (PMC11003397; doi:10.1039/d3dt01984e)
Supplement: DT-052-D3DT01984E-s001 [file DT-052-D3DT01984E-s001.pdf]

Electronic Supplementary Material (ESI) for Dalton Transactions.
This journal is © The Royal Society of Chemistry 2023

ELECTRONIC SUPPLEMENTARY INFORMATION

Selective cycloaddition of ethylene oxide to CO_2 within the confined space of an amino acid-based metal-organic framework

Cristina Bilanin,[‡a] Paula Escamilla,[‡b] Jesús Ferrando-Soria,[*b] Antonio Leyva-Pérez,[*a] Donatella Armentano[*c] and Emilio Pardo[*b]

[a] *Instituto de Tecnología Química, Universitat Politècnica de València – Consejo Superior de Investigaciones Científicas, Av .de los Naranjos, s/n, 46022 Valencia, Spain.*

[b] *Instituto de Ciencia Molecular (ICMol), Universidad de Valencia, 46980 Paterna, Valencia, Spain.*

[c] *Dipartimento di Chimica e Tecnologie Chimiche, Università della Calabria, 87036, Cosenza, Italy.*

Corresponding authors: jesus.ferrando@uv.es anleyva@itq.upv.es,
donatella.armentano@unical.it, emilio.pardo@uv.es

Phone: +34963877812; Fax: +349638 77809.

TABLE OF CONTENTS:

- Experimental section: S3
  - General S3
  - Physical characterization techniques S3
  - Procedures for the synthesis of MOF S4
  - X-Ray crystallographic section S4
  - Catalytic reaction procedures S5
  - Catalytic products characterization S6
- Table S1 and Figs. S1-S13 S8
- References S22

Experimental Section.

General.

Reagents were obtained from commercial sources and used without further purification unless otherwise indicated. Anhydrous solvents were obtained from a resin-exchanger apparatus. Reactions were performed in conventional round-bottomed flasks or sealed vials equipped with a magnetic stirrer. All the products were characterized by gas chromatography-mass spectrometry (GC-MS), proton (^{1}H), carbon (^{13}C) and distortionless enhancement by polarization transfer (DEPT) nuclear magnetic resonance (NMR) spectroscopy. Gas chromatographic analyses were performed in an instrument equipped with a 25 m capillary column of 1 % phenylmethylsilicone using *n*-dodecane as an external standard. GC/MS analyses were performed on a spectrometer equipped with the same column as the GC and operated under the same conditions. ^{1}H-, ^{13}C and DEPT were recorded in a 300 MHz (or 400 MHz when available) instrument using CD_3CN as solvent unless otherwise indicated, containing TMS as internal standard. Infrared (IR) spectra of the compounds were recorded with a spectrophotometer by impregnating the windows with a dichloromethane solution of the compound and leaving evaporate before analysis. H_2Me_2-(*S*,*S*)-Mecysmox was prepared following a reported procedure.[S1]

Elemental (C, H, S, N) analysis was performed at the Microanalytical Service of the Universitat de València. FT–IR spectra were recorded on a Perkin-Elmer 882 spectrophotometer as KBr pellets. The thermogravimetric analysis was performed on crystalline samples under a dry N_2 atmosphere with a Mettler Toledo TGA/STDA 851e thermobalance operating at a heating rate of 10 ºC min^{-1}.

Gas adsorption

The N_2 adsorption-desorption isotherms at 77 K were carried out on polycrystalline samples of **1** before and after catalysis, with a BELSORP-mini-X instrument. Samples were first activated with methanol and then evacuated at 348 K during 19 hours under 10^{-6} Torr prior to the analysis.

X–ray Powder Diffraction Measurements

Polycrystalline samples of **1** before and after catalysis were introduced into 0.5 mm borosilicate capillaries prior to being mounted and aligned on a Empyrean PANalytical

powder diffractometer, using Cu Kα radiation (λ = 1.54056 Å). For each sample, five repeated measurements were collected at room temperature (2θ = 2–45°) and merged in a single diffractogram.

Procedures for the synthesis of MOF 1.

{$Sr^{II}Zn^{II}_6$[(*S*,*S*)-Mecysmox]$_3$(OH)$_2$(H_2O)}} · 9H_2O (1): H_2Me_2-(*S*,*S*)-Mecysmox (4.22 g, 12.0 mmol) was suspended in 80 mL of water and treated with a 25% methanolic solution of Me_4NOH (14.5 mL, 50.0 mmol) until complete dissolution. Then, another aqueous solution (40 mL) containing $Sr(NO_3)_2$ (0.85 g, 4.0 mmol) and $Zn(NO_3)$ · $6H_2O$ (6.98 g, 24.0 mmol) was added dropwise under stirring. After further stirring for 10 h, at room temperature, a white polycrystalline powder was obtained and collected *via* filtration and dried with methanol. Yield: 4.83 g, 73%; Anal.: calcd for $C_{30}H_{58}N_6O_{30}S_6SrZn_6$ (1655.1): C, 21.77; H, 3.53; S, 11.62; N, 5.08%. Found: C, 21.71; H, 3.50; S, 11.65; N, 5.07%. IR (KBr): ν = 1602 cm^{-1} (C=O). Well-shaped hexagonal prisms of **1** suitable for X-ray structural analysis could be obtained by slow diffusion, in an H-shaped tube, of H_2O/DMF (1:1) solutions containing stoichiometric amounts of H_2Me_2-(*S*,*S*)-Mecysmox (0.021 g, 0.06 mmol) and Me_4NOH (0.72 mL, 2.5 mmol) in one arm and $Sr(NO_3)_2$ (0.042 g, 0.2 mmol) and $Zn(NO_3)$ · $6H_2O$ (0.035 g, 0.12 mmol) in the other. They were isolated by filtration on paper and air-dried. Anal.: calcd for $C_{30}H_{58}N_6O_{30}S_6SrZn_6$ (1655.1): C, 21.77; H, 3.53; S, 11.62; N, 5.08%. Found: C, 21.73; H, 3.49; S, 11.58; N, 5.06%.

X-ray crystallographic data collection and structure refinement.

Crystal of **1** with 0.18 x 0.18 x 0.14 mm as dimensions was selected and mounted on a MiTeGen MicroMount in Paratone oil and very quickly placed on a liquid nitrogen stream cooled at 100 K, to avoid the possible degradation upon dehydration or exposure to air. Diffraction data were collected on a Bruker-Nonius X8APEXII CCD area detector diffractometer using graphite-monochromated Mo-K_α radiation (λ = 0.71073 Å), The data were processed through SAINT[S2] reduction and SADABS[S3] multi-scan absorption software. The structure was solved with the SHELXS structure solution program, using

the Patterson method. The model was refined with version 2018/3 of SHELXL against F^2 on all data by full-matrix least squares.[S4]

In the refinement, all non–hydrogen atoms of the MOF net, except the highly thermal disordered terminal methyl of the methylcysteine thioether chains from the Mecysmox ligand, were refined anisotropically. The use of some C-C and C-S bond lengths restrains during the refinement have been reasonable imposed and related to extraordinary flexibility of methyl thioether chains from the methylcysteine residues, which are dynamic components of the framework. The disorder has been analyzed starting at the beginning of the chains, thus from C1S and C3S and using restrains on lengths (DFIX and SADI) and displacements parameters (SIMU and DELU commands). Disordered sites for atoms S2 and C4S in refinement, belonging to the methylcysteine ligand, result disordered and have been refined on two different sites (S2 and S2'; C4S and C4S') with 50:50 of occupancy factors. In this way, we modelled the most disordered chain as both dynamic and statistically disordered. In so doing, the set of data unveiled that it is still present an electron density maximum at 1.65 and 1.41 Å far from S2 and S2' sites, respectively. Considering the chemical aspects of the compound, its preparation and its crystal growth pathway, it cannot represent any reliable further atom site from chain itself. But it should be likely related to both the intrinsic flexibility of the chain and solvent disordered in pores. Of course, assigning the detected density as a fully occupied O_{water} atom would be, according to us, an artefact related to the flexibility of the system and, chemically speaking, a worst interpretation of diffraction data, if not considering it as a pseudo-superimposed situation due to plausible disorder. Disordered water molecules might reside in sites otherwise occupied by methylcysteine fragments. The latter can adopt different conformations and only one of that, likely the most populated one, has been "averaged" in the proposed view related to its orientations in a single unit cell.

All the hydrogen atoms of the network were set in calculated position and refined isotropically using the riding model except for those belonging to the most disordered chain.

The thermal disorder of fragments pointing towards the pores is likely related to the porosity of the network.

The solvent molecules, as normally observed for such porous crystals, were highly disordered and only few of them were found from the ΔF map. The quite large channels featured by this series of MOFs likely account for that.

A summary of the crystallographic data and structure refinement for **1** crystal structure is given in Table S1. The comments for the alerts A and B are described in the CIF using the validation reply form (vrf). CCDC reference number is 2265848.

The final geometrical calculations on free voids and the graphical manipulations were carried out with PLATON[S5] implemented in WinGX,[S6] and CRYSTAL MAKER[S7] programs, respectively. To the experimental set of data, has been applied a cut-off with 2theta of 56°.

Catalytic reaction procedures.

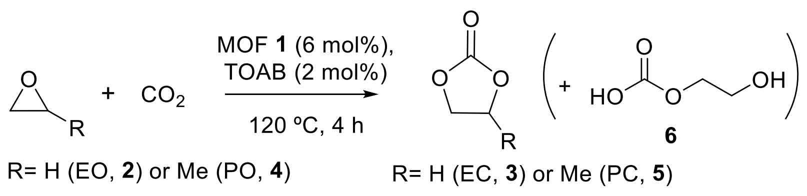

General procedure. Products **3**, **5** and **6** were prepared following the reaction scheme. Catalyst **1** (0.06 eq, 0.16 mmol), reactants **2** or **4** (1 eq, 2.72 mmol) and TOAB (0.02 eq, 0.058 mmol) in 1.7 mL of dry THF were introduced in a reactor equipped with a magnetic stirrer. Then, the reactor was closed, was purged with N_2 two times and carbon dioxide (25 bar) was introduced. The reaction was magnetically stirred in a pre-heated oil bath at

120 ºC during typically 4 h. After that time, the resulting mixture was filtered and analyzed by GC, GC-MS and NMR.

Hot filtration test. Following the general reaction procedure above, the hot reaction mixture was filtered at 30 minutes when the reaction conversion is *ca*. 20%. Filtrates were placed into a reactor, which was closed and purged with N_2 twice, and carbon dioxide (25 bar) was introduced. Finally, the reaction is placed at the reaction temperature (120 ºC). The filtrates were periodically analysed by GC, comparing the results obtained with the solid catalyst still in.

Reuses. When the reaction is finished, the solid is separated by centrifugation and washed with THF (three times) to remove the ammonium salt and any soluble product. Then, the solid catalyst is dried under vacuum, weighted, and directly used in the next reaction.

Catalytic products characterization.

Ethylene carbonate **3**. GC-MS (m/z, $M^{+\bullet}$ 88), major peaks found: 88, 58, 43, 29, 15. ^{1}H NMR (400 Hz, $CDCl_3$) δ 4.51 (s, 4H). FT-IR 1767-1788 (C=O); 1217-1231 (C-O).

Propylene carbonate **5**. GC-MS (m/z, $M^{+\bullet}$ 102), major peaks found: 102, 87, 57, 43, 39, 29, 15. ^{1}H NMR (300 MHz, $CDCl_3$) δ 4.85 (m, 1H), 4.55 (dd, J = 8.4, 7.7 Hz, 1H), 4.02 (dd, J = 8.4, 7.2 Hz, 1H), 1.49 (d, J = 6.3 Hz, 3H). FT-IR 1782 (C=O); 1175 (C-O).

2-Hydroxyethyl hydrogen carbonate **6**. GC-MS (m/z, $M^{+\bullet}$ 106), major peaks found: 106, 86, 71, 56, 42, 28, 18.

2,2-Bis(ethylthio)-1,3-dioxolane **7**. MS (m/z, [$M^{\bullet}$ -H] 193), major peaks found: 193, 178, 150, 134, 106, 75, 58, 47, 27, 17. ^{1}H NMR (300 MHz, $CDCl_3$) δ 4.51 (s, 4H), 2.54 (q, J = 7.4 Hz, 2H), 1.25 (t, J = 7.4 Hz, 3H). FT-IR 1801-1774 (C-S); 1160 (C-O).

Table S1. Summary of crystallographic data for **1**

Compound	**1**
Formula	$C_{30}H_{58}SrCu_6N_6O_{30}S_6$
M (g mol^{-1})	1655.02
λ (Å)	0.71073
Crystal system	hexagonal
Space group	$P6_3$
a (Å)	18.5594(15)
c (Å)	11.7771(13)
V (Å^3)	3513.1(7)
Z	2
ρ_{calc} (g cm^{-3})	1.565
μ (mm^{-1})	3.019
T (K)	90
θ range for data collection (°)	2.534 to 26.415
Completeness to θ = 25.0	100%
Measured reflections	50455
Unique reflections (Rint)	4809 (0.0881)
Observed reflections [$I > 2\sigma(I)$]	2939
Goof	1.019
Absolute structure parameter (Flack)	0.14(2)
R^a [$I > 2\sigma(I)$] (all data)	0.0597 (0.1196)
wR^b [$I > 2\sigma(I)$] (all data)	0.1571(0.1909)
CCDC	2265848

^{a} $R = \sum(|F_o| - |F_c|)/\sum|F_o|$. ^{b} $wR = [\sum w(|F_o| - |F_c|)^2/\sum w|F_o|^2]^{1/2}$.

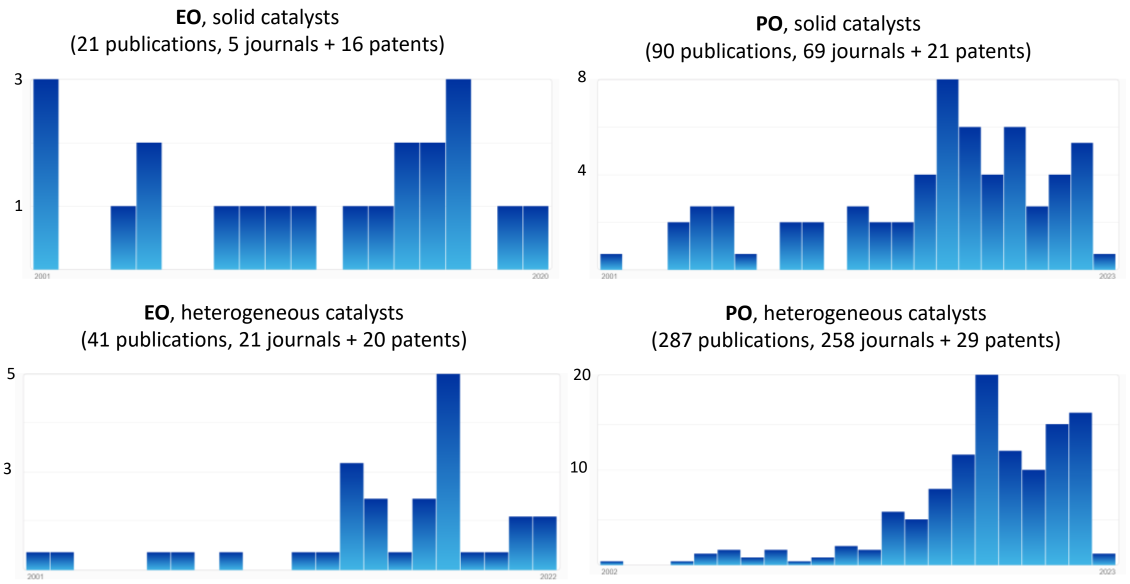

Fig. S1. Reported studies on the cycloaddition reaction of either ethylene oxide (EO, left) or ethylene oxide (PO, right) to CO_2 to give ethylene carbonate (EC) or propylene carbonate (PC), respectively, with solid (top) or generally speaking heterogeneous catalysts (bottom). Source: Scifinder^{n,TM}, searching for the specific reaction and then truncating with the term above detailed.

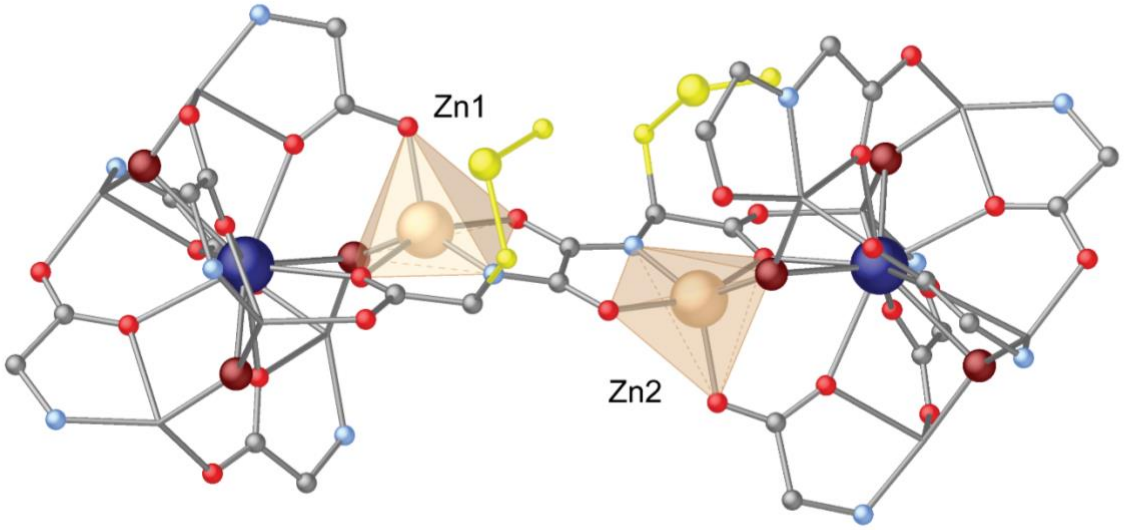

Fig. S2. View of the dianionic bis(hydroxo)dizinc(II) building blocks in **1**, highlighting with polyhedra the pentacoordinated Zn(II) ions. Zn(II), Sr(II) ions and water molecules/hydroxide ions are displayed as salmon polyhedral, dark blue and big red spheres, respectively. The carbon, oxygen, nitrogen and sulphur from the ligand are depicted as grey, red, sky-blue and yellow small spheres.

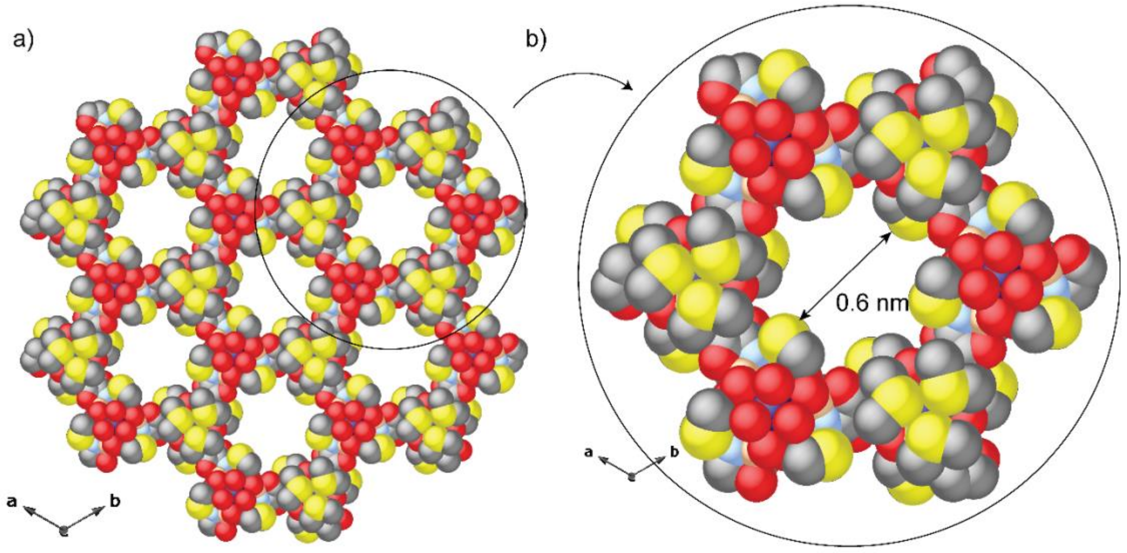

Fig. S3. a) View of 3D network of **1** and b) details of one single pore along *c* crystallographic axis showing the porous structure in space filling model (van deer Waals radii). Zn(II) and Sr(II) metal ions are represented by salmon and blue spheres, respectively, whereas the oxygen, carbon, nitrogen and sulphur atoms from the ligands are depicted as red, grey, light blue and yellow spheres.

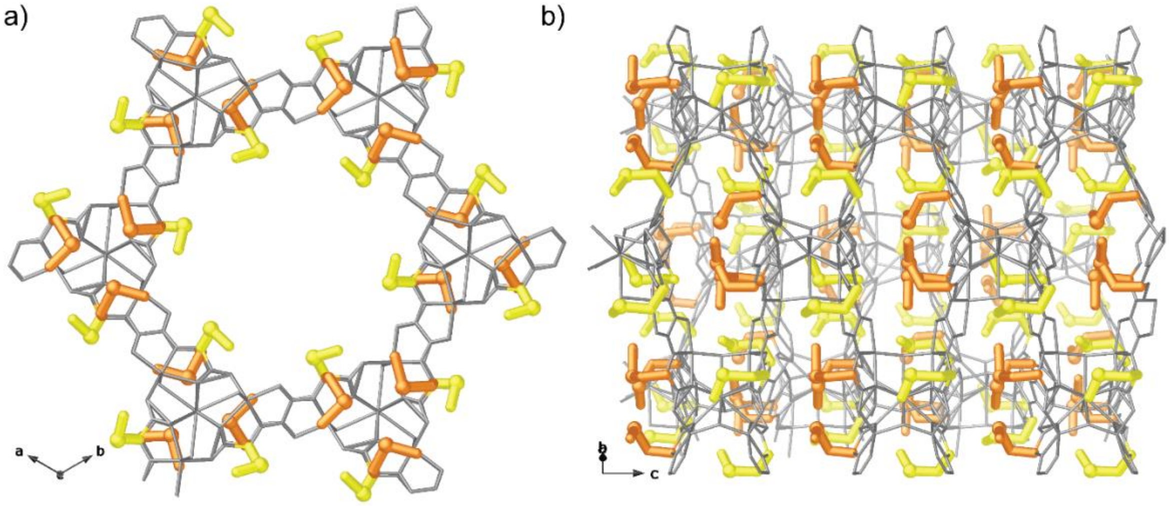

Fig. S4. Perspective view of one single channel of **1** along the *c* (a) and *a* (b) axes. Zn, Sr and organic ligands are represented as thin grey sticks for the sake of clarity and to emphasise the amino acid residues. In turn, the more distended and more bent -CH_2SCH_3 chains (amino acid residues) are represented as thick yellow and orange sticks, respectively

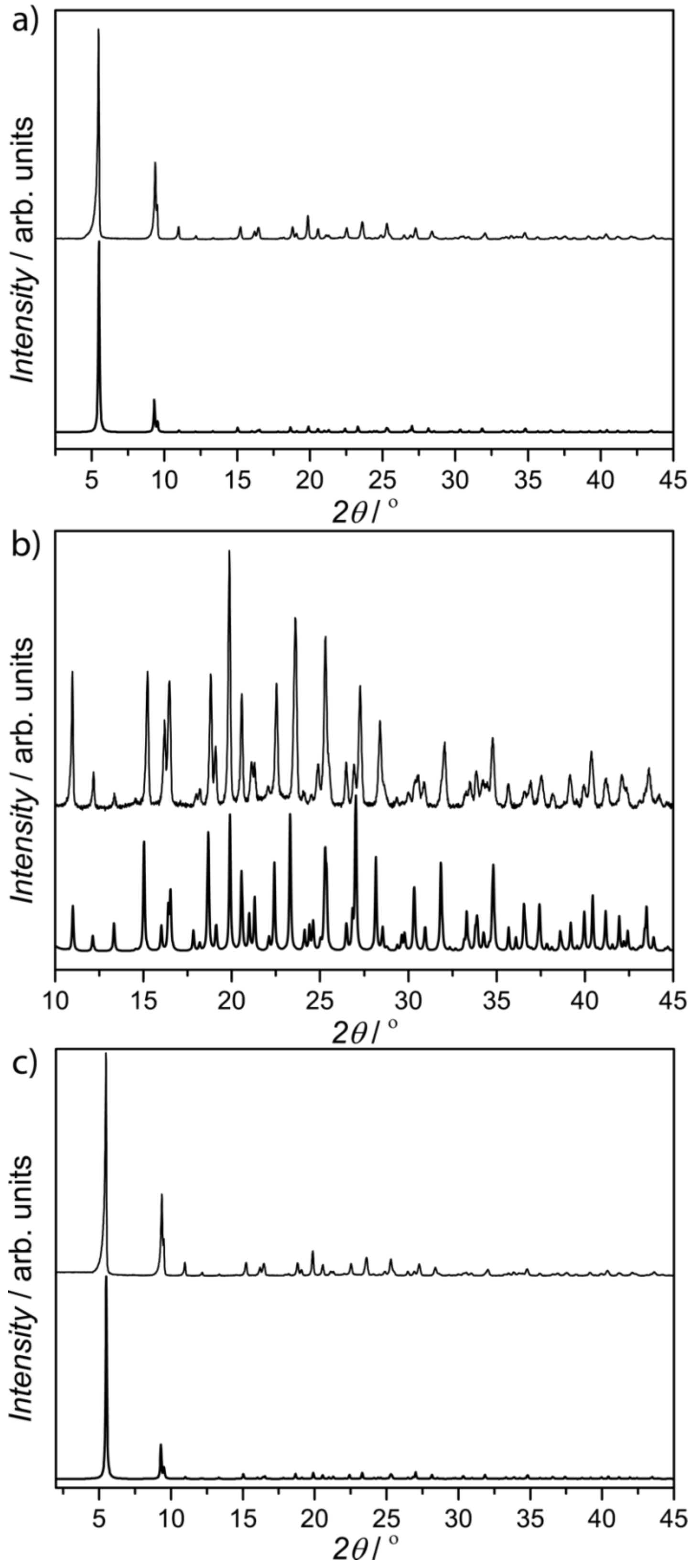

Fig. S5. (a) Calculated (bold lines) and experimental (solid lines) PXRD pattern profiles of **1** in the 2θ range 2.0–45.0° at room temperature, and enlarged image in the range 10.0–45.0° (b). (c) Calculated (bold lines) and experimental (solid lines) PXRD pattern profile of **1** after five uses in the catalytic cycloaddition reaction of ethylene oxide (EO **2**), in the range 2.0–45.0°.

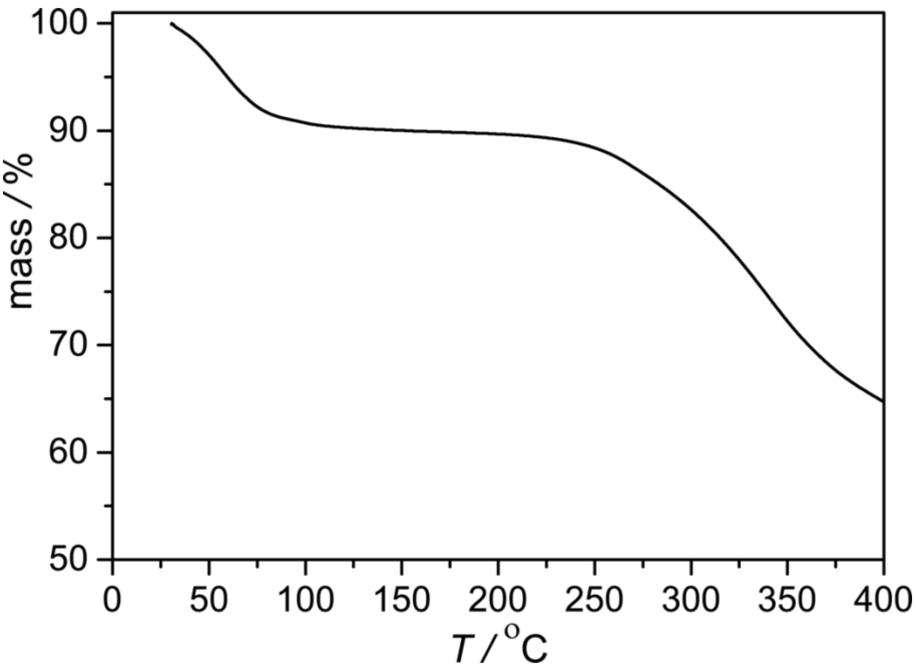

Fig. S6. Thermo-Gravimetric Analyses (TGA) of **1** under dry N_2 atmosphere.

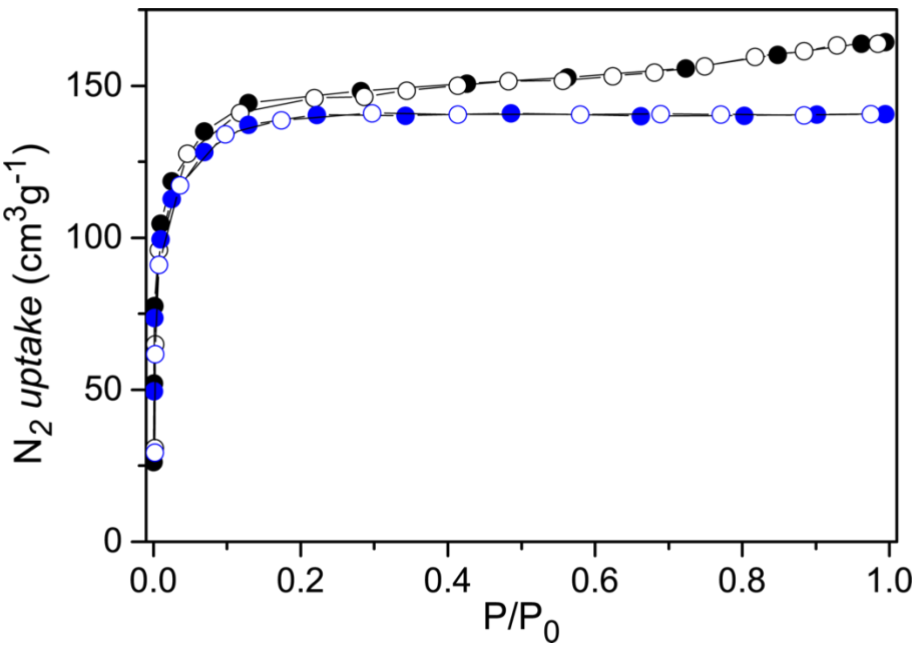

Fig. S7. N_2 (77 K) adsorption isotherm for the activated compounds **1** before (black) and after catalysis (blue). Filled and empty symbols indicate the adsorption and desorption isotherms, respectively. The samples were activated at 348 K under reduced pressure for 19 h prior to carry out the sorption measurements.

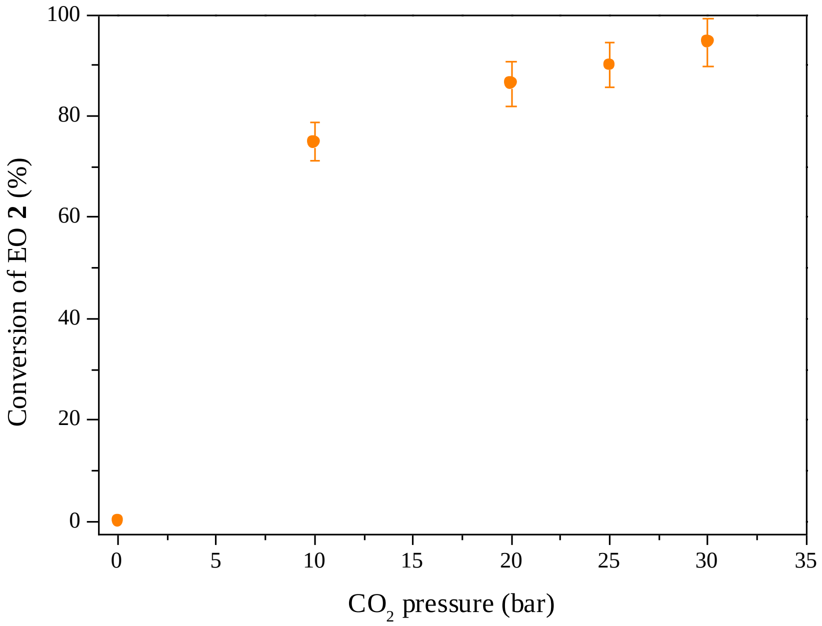

Fig. S8. Results for the cycloaddition reaction of ethylene oxide (EO **2**) dissolved in THF (1.6 M), to give ethylene carbonate (EC **3**) with catalytic amounts of MOF **1** (6 mol%) and TOAB (2 mol%) under increasing CO_2 pressures at 120 ºC reaction temperature. Selectivity is 100%. Error bars account for a 5% uncertainty.

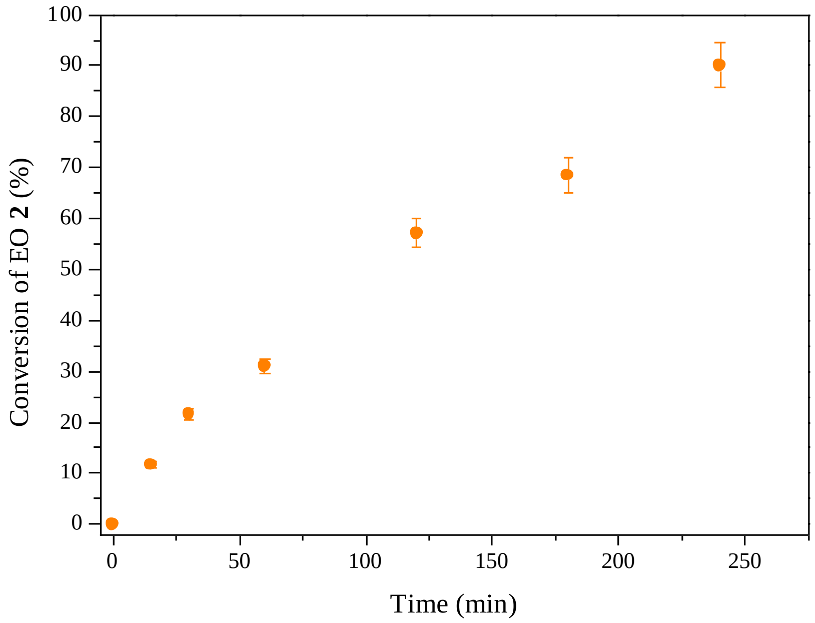

Fig. S9. Kinetic plot for the cycloaddition reaction of ethylene oxide (EO **2**) dissolved in THF (1.6 M), to give ethylene carbonate (EC **3**) with catalytic amounts of MOF **1** (6 mol%) and TOAB (2 mol%) under 25 bars of CO_2 at 120 ºC reaction temperature. Selectivity is 100%. Error bars account for a 5% uncertainty.

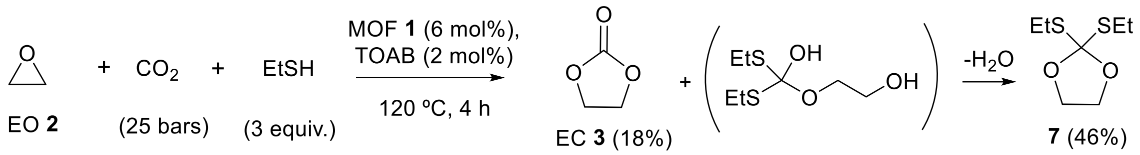

Fig. S10. Cycloaddition reaction of ethylene oxide (EO **2**) dissolved in THF (3.3 M) in the presence of EtSH (3 equivalents), to give ethylene carbonate (EC **3**) and thioketal **7**, with catalytic amounts of MOF **1** (6 mol%) and TOAB (2 mol%) under 25 bars of CO_2 at 120 ºC for 4 h.

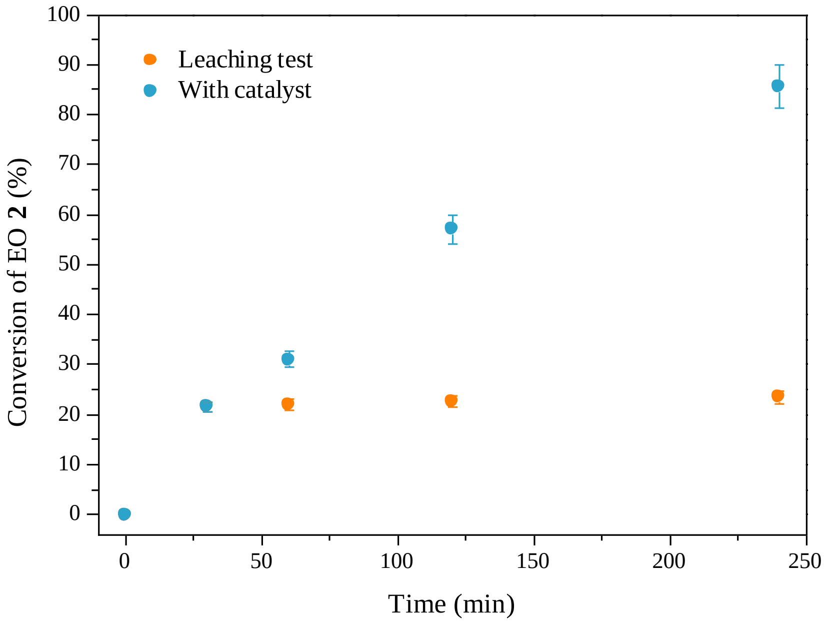

Fig. S11. Hot filtration test for the cycloaddition reaction of ethylene oxide (EO **2**) dissolved in THF (3.3 M) to give ethylene carbonate (EC **3**), with catalytic amounts of MOF **1** (6 mol%) and TOAB (2 mol%) under 25 bars of CO_2 at 120 ºC. Error bars account for a 5% uncertainty.

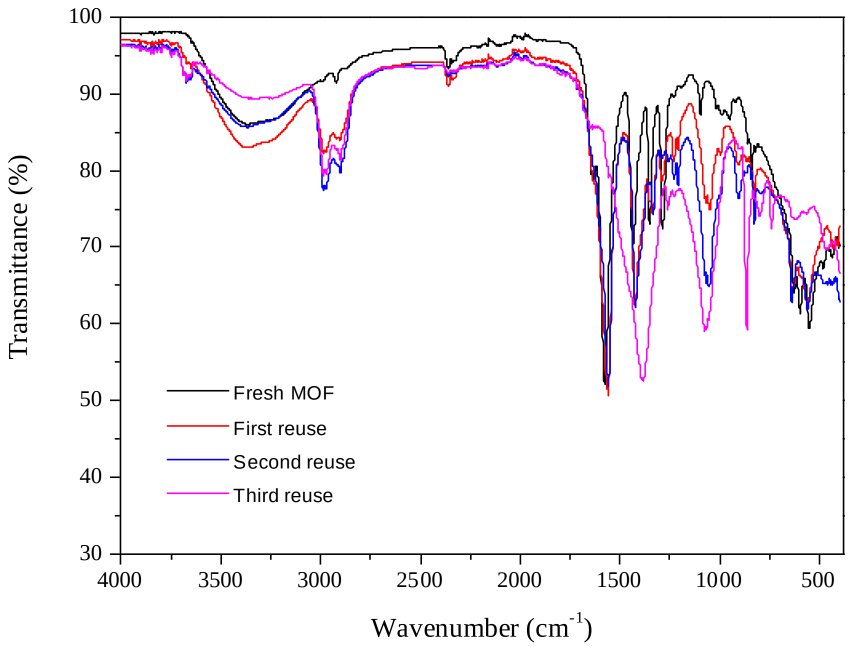

Fig. S12. Fourier-transformed infrared (FT-IR) spectra of MOF **1** (black line) and after several uses.

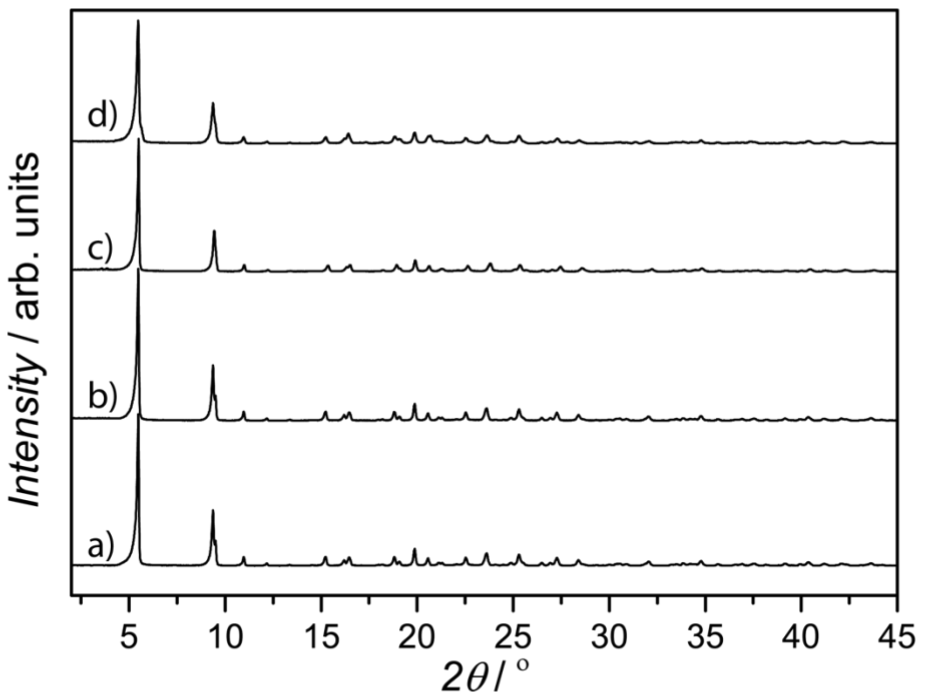

Fig. S13. Experimental PXRD pattern profiles of **1** in the form of polycrystalline powder (a), and after 24 hours immersed in normal water (b) and water at pH = 5 (c) and 11 (d) in the 2θ range 2.0–45.0° at room temperature.

References

[S1] R. Bruno, M. Mon, P. Escamilla, J. Ferrando-Soria, E. Esposito, A. Fuoco, M. Monteleone, J. C. Jansen, R. Elliani, A. Tagarelli, D. Armentano and E. Pardo, Bioinspired Metal-Organic Framework in Mixed Matrix Membranes for Efficient Static/Dynamic Removal of Mercury from Water, *Adv. Funct. Mater.*, 2020, 2008499.

[S2] SAINT, version 6.45, Bruker Analytical X-ray Systems, Madison, WI, 2003.

[S3] Sheldrick G.M. SADABS Program for Absorption Correction, version 2.10, Analytical X-ray Systems, Madison, WI, 2003.

[S4] (a) Sheldrick, G. M. Crystal structure refinement with SHELXL. *Acta Cryst. C* **71**, 3 (2015). (b) Sheldrick, G. M. A short history of SHELX. *Acta Cryst. A* **64**, 112 (2008). (c) SHELXTL-2013/4, Bruker Analytical X-ray Instruments, Madison, WI, 2013.

[S5] (a) Spek, A. L. *PLATON* SQUEEZE: a tool for the calculation of the disordered solvent contribution to the calculated structure factors. *Acta Crystallogr. Sect. C-Struct. Chem*. 2015, **71**, 9–18. (b) Spek, A. L. Structure validation in chemical crystallography. *Acta Crystallogr. Sect. D, Biol. Crystallogr.* 2009, **65**, 148–155.

[S6] Farrugia, L. J. *WinGX* suite for small-molecule single-crystal crystallography. *J. Appl. Crystallogr*. 1999, **32**, 837–838.

[S7] Palmer, D. CRYSTAL MAKER, Cambridge University Technical Services, C. No Title, 1996.
